# Epidemiological survey of sheep as potential hosts for *Leishmania* in China

**DOI:** 10.1186/s12917-018-1701-z

**Published:** 2018-12-03

**Authors:** Shuai Han, Wei-Ping Wu, Kai Chen, Israyil Osman, Kaisar Kiyim, Jun Zhao, Yan-Yan Hou, Ying Wang, Li-Ying Wang, Can-Jun Zheng

**Affiliations:** 1 National Institute of Parasitic Diseases, Chinese Center for Disease Control and Prevention, WHO Collaborating Center for Tropical Diseases, Key Laboratory of Parasite and Vector Biology, Ministry of Health, National Center for International Research on Tropical Diseases, Ministry of Science and Technology, Shanghai, 200025 China; 2 Xinjiang Uygur Autonomous Regional Center for Disease Control and Prevention, Urumqi, China; 3 Kashgar Prefectural Center for Disease Control and Prevention, Kashgar, China; 4 Chinese Center for Disease Control and Prevention, Beijing, China

**Keywords:** *Leishmania*, Visceral leishmaniasis, China, Phylogenetics, Sheep, Reservoir

## Abstract

**Background:**

*Leishmania* parasites cause visceral leishmaniasis (VL), an important infectious disease that is endemic to large parts of the world and often leads to epidemics. Sand flies are the primary transmission vector for the parasite in endemic regions. We hypothesized that sheep might serve as an overlooked reservoir for *Leishmania* transmission to humans due to the asymptomatic nature of infection in many species. As a preliminary test of this hypothesis, the aim of the present study was to investigate sheep in an area of China that is endemic for the desert sub-type of zoonotic VL and establish if they are potential carriers of *Leishmania*.

**Results:**

Sheep tissue samples were collected from abattoirs in VL endemic areas of Jiashi County, China during the non-transmission season. rK39 immunochromatographic tests were performed to detect the presence of the parasite in blood samples. In addition, DNA was extracted from the blood, and used for detection of the *Leishmania*-specific internal transcribed spacer-1 (ITS-1) genomic region using a nested polymerase chain reaction (PCR) approach. PCR products were further analyzed to identify restriction fragment-length polymorphism patterns and representative sequences of each pattern were selected for phylogenetic analysis. The rK-39 and nested PCR data indicated positive detection rates for *Leishmania* in sheep of 26.32 and 54.39%, respectively. The phylogenetic analysis revealed that all of the samples belonged to the species *L. infantum* and were closely related to strains isolated from human infections in the same area*.*

**Conclusions:**

Sheep could be a potential host for *Leishmania* in VL endemic areas in China and may be an overlooked reservoir of human VL transmission in this region. To further confirm livestock as a potential host, further verification is required using a sand fly biting experiment.

## Background

Kala-azar, also known as visceral leishmaniasis (VL), is a parasitic disease caused by various *Leishmania* species and is endemic in many countries. Sand flies are the main transmission vector for the disease. The primary clinical features of VL include long-term irregular fevers, splenomegaly, anemia, emaciation, leukopenia, and an increase in serum globulin levels. Most untreated VL patients die within 2 years of contracting the illness due to related complications [1–3]. Approximately 200,000–400,000 new cases of VL are reported annually, with more than 90% found in India, Bangladesh, Sudan, South Sudan, Ethiopia, and Brazil [4, 5]. In China, VL is characterized according to differences in endemic area, pathogen species, and vector species into anthroponotic VL, a mountain sub-type of zoonotic VL, and a desert sub-type of zoonotic VL [6, 7]. The desert sub-type of zoonotic VL is specifically caused by *Leishmania infantum* [8, 9] and is most prevalent in Minfeng, the Bachu Reclamation Regions, the eastern regions of Jiashi in Xinjiang, Ejin Banner in Inner Mongolia, and Dunhuang in Gansu [10–12]. The desert sub-type of zoonotic VL is highly endemic to Jiashi County of Kashgar Prefecture in Xinjiang which is located near the central and lower reaches of the Tian Shan mountain range floodplain in the western margins of the Tarim Basin [13, 14]. Most patients with this sub-type are infants under 2 years of age and the mortality rate can reach 95% or higher if left untreated [15]. In 2007, only 19 cases of VL were reported in Jiashi County; however, there were 214 reported cases in 2008, largely due to the fact VL is often neglected and its severity is thus underestimated [16–18]. Moreover, accumulating evidence in recent years points to a continuing upward trend of leishmaniasis cases in the region, suggesting an epidemic outbreak [19].

Gaining a detailed understanding of this situation is further complicated by the fact that humans and animals infected with *Leishmania* are often asymptomatic or show latent infections. These asymptomatic hosts may therefore play an important role in the transmission of the parasite [20–23]. Based on previous evidence, it has been proposed that natural foci exist within the desert sub-type of zoonotic VL endemic areas and that wild animals may serve as hosts [24–28]. Although the local residents of this area do not tend to keep dogs as pets, wild rodents and other domestic animals have been considered to be potential hosts for Leishmania in other regions [29, 30]. Accordingly, since sheep are by far the most common livestock animal in Jiashi County, we hypothesized that sheep might be an overlooked reservoir of *Leishmania* in this region, contributing to the increasing incidence of leishmaniasis. Importantly, the small number of VL cases in China suggests that the disease could feasibly be eliminated if appropriate control strategies are implemented. However, the main source of VL infection in the desert sub-type of zoonotic VL endemic areas is still unknown, thereby hindering such efforts [25, 27, 31]. In addition, there are currently no strategies or programs in place to control the disease, except for treating patients on a case-by-case basis. Therefore, identifying the primary host species for *Leishmania* in China would be an important step towards the prevention of VL. A critical tool that can be used for such analysis is *Leishmania-*specific polymerase chain reaction (PCR) targeting the internal transcribed spacer 1 (ITS-1) region between the genes encoding the SSU and 5.8S rRNAs. [32, 33]. To identify potential sources of VL transmission in China, we used both rK39 immunochromatographic tests and ITS-1 nested PCR to examine *Leishmania* infection rates in tissue samples from sheep collected in endemic areas of the desert sub-type of zoonotic VL. We also performed phylogenetic analysis and compared the strains isolated from sheep with those retrieved from human patients in the same area.

## Results

### Sheep *Leishmania* infection rate determined using rK39 assays

A total of 114 sheep were investigated in this survey, including 99 males and 15 females. The positive detection rate of sheep blood samples was 26.32% (30/114), determined using the rK39 immunochromatographic strip test (Table 1). There were no significant differences between positive detection rates in sheep of different dental ages (*P* > 0.05, χ^2^ test or Fisher’s exact test were applied to compare data between genders within dental ages and the total) (Table 1).

**Table 1:** Relationship among VL infection, gender, and dental age of sheep, detected using the rK39 test

Dental Age (months)	Male Sheep			Female Sheep			Statistical test	
	Number	Number of Positive	Positive rate	Number	Number of Positive	Positive rate	χ^2^	*P* value
~ 2	70	19	27.14%	6	2	33.33%	9.07E-31	1.00
~ 4	20	5	25.00%	0	0	–	–	–
~ 6	9	2	22.22%	9	2	22.22%	–	1.00
Total	99	26	26.26%	15	4	23.67%	2.14E-31	1.00

*PCR* Polymerase chain reaction

### Sheep *Leishmania* infection rate determined using nested PCR

**Fig. 1:**
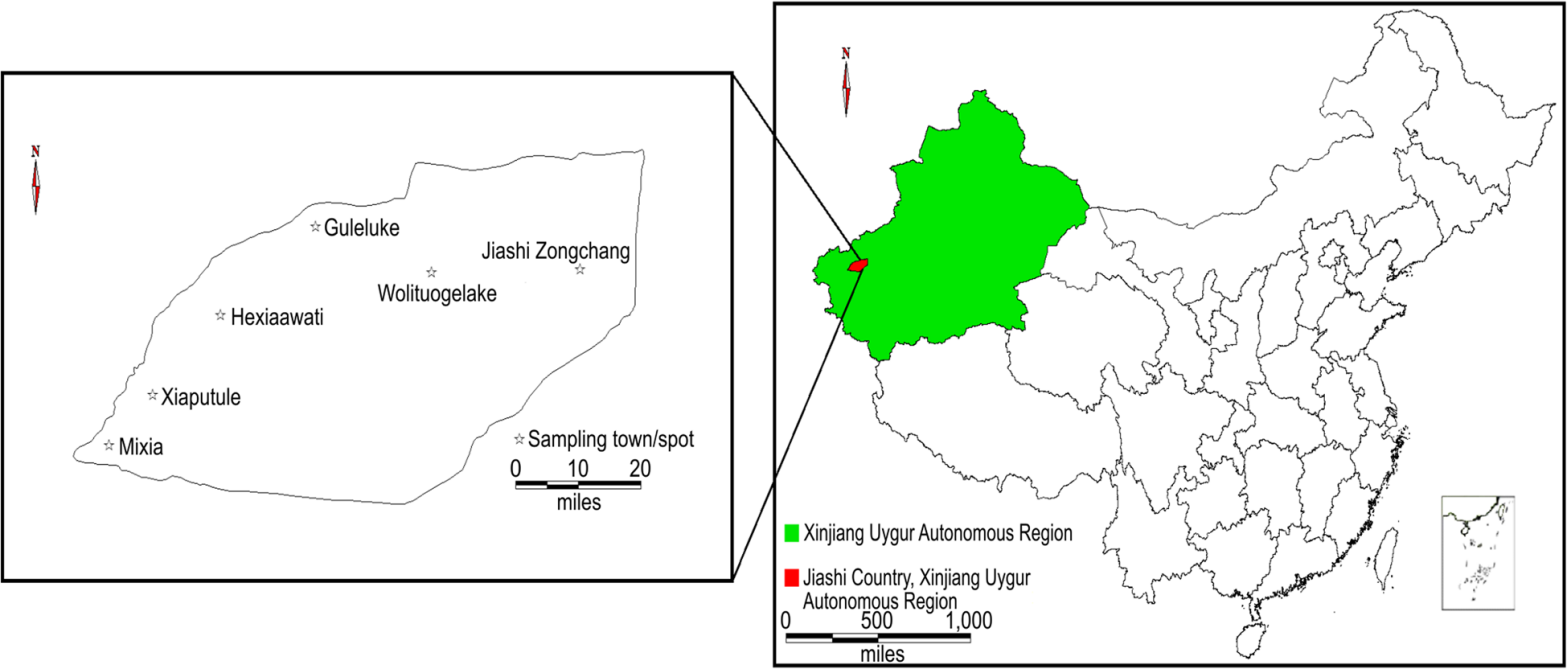
Nested PCR amplification of the ITS-1 region in sheep. M: 50 bp DNA marker; 101–118: whole sheep blood DNA as a template; LS: positive control; NC: negative control

The 285-bp bands representing the ITS-1 target sequence of *Leishmania* were successfully amplified in many of the nested PCRs (Fig. 1), and no bands of other sizes were detected. The positive infection rate of *Leishmania* across all sheep was 54.39% (62/114) using this method (Table 2). Again, no significant differences were found in the positive detection rates among sheep of different dental ages (P > 0.05, χ^2^ test or Fisher’s exact test as appropriate to compare data between genders within dental ages and the total) (Table 2).

**Table 2:** Relationship among VL infection, gender, and dental age of sheep, detected using nested PCR tests

Dental Age (months)	Male Sheep			Female Sheep			Statistical test	
	Number	Number of Positive	Positive rate	Number	Number of Positive	Positive rate	χ2	*P* value
~ 2	70	40	57.14%	6	3	50%	0.00	1.00
~ 4	20	10	50.00%	0	0	–	–	–
~ 6	9	3	33.33%	9	6	66.67%	–	0.35
Total	99	53	53.54%	15	9	60%	0.04	0.85

*PCR* Polymerase chain reaction

### RFLP analysis

**Fig. 2:**
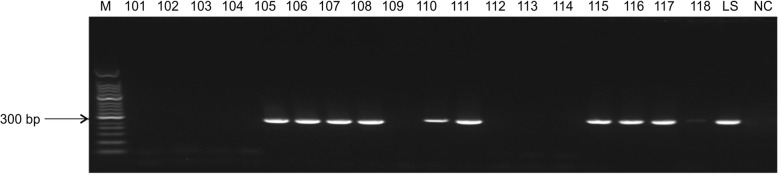
*Hae*III restriction map of nested ITS-1 PCR from sheep samples. M: 50 bp DNA marker; 32–51: whole sheep blood DNA as a template; LS: positive control; NC: negative control

The *Hae*III restriction maps of all positive ITS-1 nested PCR products were determined to be identical, with bands 161 bp, 69 bp, and 55 bp in size (Fig. 2). This indicated that the target bands all originated from genetically similar parasite strains.

### Phylogenetic analysis

**Fig. 3:**
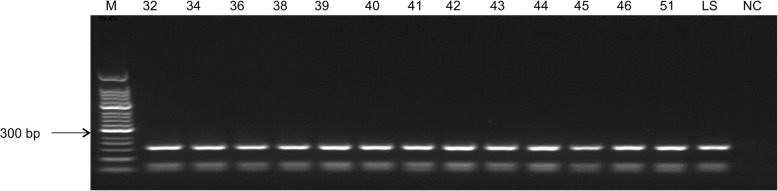
Phylogenetic analysis of the ITS-1 sequence obtained from positive sheep. LS-36: sheep tissue samples; XJR-25: VL patient blood samples; LS-Y (MHOM/CN/08/JS-1): positive control

As shown in Fig. 3, the target sequences (i.e., those most closely related to the LS-36 strain, identified in this study) isolated from the sheep blood samples (GenBank:KT153645) and LS-Y (the positive control *Leishmania* strain MHOM/CN/08/JS-1, GenBank:KT153649) clustered in the same phylocluster. This suggests that the local sheep were infected with *L. infantum*. Importantly, LS-36 clustered together with sequences obtained from blood samples isolated from human VL patients from Jiashi County (XJR-25, GenBank:KT153646).

## Discussion

Some studies have suggested that asymptomatic infected humans may play an important role in the spread of anthroponotic Leishmania donovani, although further verification by xenodiagnosis using the competent phlebotomine vector Phlebotomus argentipes is required [21, 22, 34, 35]. Given the equally high rate of asymptomatic livestock, the possibility that asymptomatic sheep may also serve as an overlooked host for *Leishmania* in endemic areas cannot be excluded [36]. In our study area in China, wild rodents such as *Lepus yarkandensis* and asymptomatic infected humans are generally considered to be the potential hosts of *Leishmania*, and most infections have thus far been limited to infants under two years of age [15, 37]. In these endemic areas, cases of leishmaniasis are temporally isolated, with a season of high incidence between September to March and peak incidence from October to December. However, there has been a trend in recent years towards increasing *Leishmania* outbreaks [19],and several patients have become ill during the non-transmission season, although they recovered after treatment. These patients were unlikely to be a source of infection for the sand fly vector *Phlebotomus wui*, especially during the non-transmission period when the vector is not present. There are three conditions necessary for the maintenance of mediated infectious disease foci: presence of a pathogen, a transmission vector, and an appropriate host. *P. wui* has previously been determined to be the main vector for *Leishmania* in the endemic desert sub-type of zoonotic VL areas of Jiashi County. The species usually begins to appear in early May and disappears in early September, matching the high *Leishmania* transmission season [38, 39]. In our study, the samples were collected from sheep in April, which is the non-transmission season but is close to the next transmission season*.* However, we consider that the detection of *Leishmania* infection in sheep at this time is unlikely to have arisen due to *P. wui* transmission, although this could reflect sand fly infection from the previous season.

The conventional method to detect the parasites that cause VL is to use an rK39 dipstick, which is an in vitro diagnostic medical device designed for the qualitative detection of antibodies specific to members of the *L. donovani* complex in human serum [40–42]. However, this tool can only be used to identify patients with active disease and cannot detect asymptomatic carriers [43–47]. Leite et al. [48] evaluated the detection efficiencies of ITS-1 nested PCR and kDNA PCR hybridization to detect *L. infantum* infections in asymptomatic dogs, and found that the detection rate of ITS-1 nested PCR was up to 83.3% for conjunctival swabs and 56.7% for blood samples, which was higher than the13.3% detection efficiency for the kDNA PCR hybridization method. Pilatti et al. [49] also found that ITS-1 nested PCR detection rates were equally high (73.9%) for symptomatic dogs. These studies indicate that ITS-1 nested PCR has excellent detection sensitivity for both symptomatic and asymptomatic *Leishmania*-infected animals [50, 51]. We further confirmed this good detection sensitivity of *Leishmania* in this region of China with a much higher detection rate from ITS-1 nested PCR (54.39%) than obtained with the rK39 test (26.32%).These results confirm that the ITS-1 nested PCR is a highly specific and sensitive method for detecting *Leishmania* infection.

Finally, our phylogenetic analysis indicated that the sheep were specifically infected with *L. infantum*. There was a close phylogenetic relationship between the LS-36 strain identified in the sheep and the XJR-25 strain isolated from human VL patients from Jiashi County. This indicates that *Leishmania* transmission could potentially occur between sheep and humans in this area of China; however, further investigations on potential sheep infectiousness through xenodiagnosis assays using competent phlebotomine vectors (e.g. *P. wui*), are still needed to understand the transmission routes in this region.

One main caveat of the study is that although the sheep were bred and raised locally, imported sheep largely come from the Ili region of Xinjiang. The majority of imported sheep have been in Jiashi County for 0.5–1 years and our data cannot determine whether they became infected with *Leishmania* locally or arrived with the infection. However, as the Ili region is not endemic for VL, we consider it unlikely that they were infected before transfer.

## Conclusions

Our data suggest that asymptomatic sheep infected with *Leishmania* contribute to VL transmission between seasons in China. This overlooked reservoir represents an important potential source of local infection for the human population across seasons. Although further verification by *Phlebotomus argentipes* xenodiagnosis is still needed, our study suggests that consideration of asymptomatic sheep as hosts will be crucial for the future development of VL prevention strategies.

## Methods

### Experimental setting and collection of blood samples from sheep

**Fig. 4:**
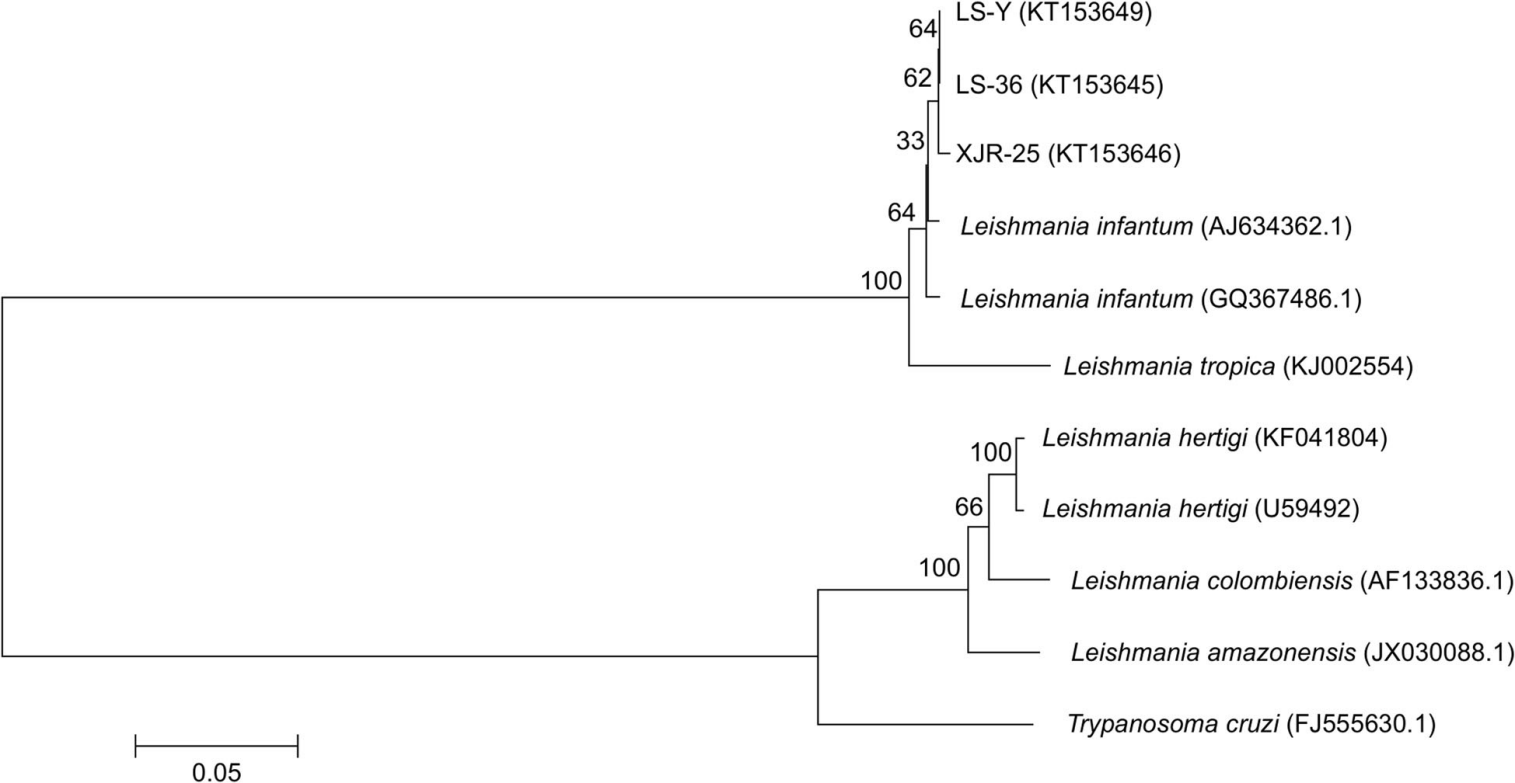
Distribution of sampling locations. A total of 31 and 10 samples of sheep were collected from the abattoirs of Wolituogelake and Jiashi Zongchang townships, respectively. A total of 49, 2, 19, and 3 samples of sheep were collected from the abattoirs of Mixia, Xiaputule, Hexiaawati, and Guleluke townships, respectively

During mid-to-late April of 2014, a total of 114 samples of approximately 3 mL blood were collected from sheep in 6-mL ethylene diamine tetraacetic acid (EDTA) blood collection tubes (BD Vacutainer; Becton Dickinson, Franklin Lakes, NJ, USA). The animals were all at least 1 year of age when slaughtered and were selected from local abattoirs with the approval and management of animal husbandry and veterinary departments in the endemic areas of Jiashi County. Sampling was performed in abattoirs of six townships across the county (Fig. 4). All samples were identified with a unique number code and stored at 4 °C until use. The study did not involve animal husbandry or sacrifice, and samples were collected from scheduled slaughters without influencing abattoir routines.

### rK39 strip tests

For the immunochromatographic identification of *Leishmania* infection, 20 μL samples of whole blood were taken and added to the sample pads of rK39 strips (InBios, Seattle, WA, USA), followed by the addition of 1–2 drops of phosphate buffered saline (PBS). Results were obtained by visually assessing the strip for the presence of bands after 5–10 min.

### DNA extraction

Total DNA was extracted from 300 μL whole blood using an SE Blood DNA kit (Omega Bio-Tek, Norcross, GA, USA), following the manufacturer’s instructions. The purified DNA was stored at − 20 °C until analysis.

### Nested PCR for *Leishmania* ITS-1 detection

Extracted DNA samples were used the template in ITS-1 nested PCR [32] with the primers indicated by Ferreira et al. [33]. Primers were synthesized by Invitrogen Trading Shanghai Co., Ltd. (Shanghai, China). An Applied Biosystems PCR thermocycler was used for all reactions (Applied Biosystems, Foster City, CA, USA). The first round of PCR was performed using a total volume of 50 μL, including 1 μL DNA template, 25 μL MAX PCR Master Mix (TaKaRa Biotechnology (Dalian) Co., Ltd., Dalian, China), 1 μL of each primer (5′-CTGGATCATTTTCCGATG-3′ and 5′-TGATACCACTTATCGCACTT-3′) at 10 μM, and ddH_2_O to reach the final volume. Positive and negative controls were also included, with DNA from *L. infantum* strain MHOM/CN/08/JS-1 as the positive control. The PCR conditions were 94 °C for 5 min; 30 cycles of 94 °C for 30 s, 53 °C for 30 s, and 72 °C for 30 s; and a final extension step at 72 °C for 5 min. The second PCR was performed in a total volume of 25 μL, including 10 μL of a 1:40 dilution of the PCR products from the first PCR as a template, 12.5 μL MAX PCR Master Mix, 1 μL each primer at 10 μM, and up to 25 μL ddH_2_O to reach the final volume. The PCR conditions were the same as those used in the first reaction. The sizes of the target fragment were between 280 and 330 bp and were detected by electrophoresis of 5 μL of the PCR products from the second reaction on a 1.5% agarose gel, visualized using a gel imager.

### Restriction fragment-length polymorphism (RFLP) analysis

ITS-1 positive nested PCR products were digested using 1 μL of the restriction endonuclease *Hae*III (TaKaRa Biotechnology (Dalian) Co., Ltd.; 50 ng/μL), 2 μL 10 × M Buffer, ≤ 1 μg DNA, and 20 μL sterilized water. The mixture was incubated at 37 °C in a water bath for 1 h. Next, 5 μL of the digested product was separated by electrophoresis on a 1.5% agarose gel to obtain restriction maps for RFLPs. If ITS-1 positive nested PCR products had the same restriction maps, they were considered to be from the same phylogenetic group. Representative samples of DNA from each group were sent to Shanghai Sangon Biological Engineering Technology & Services Co., Ltd. (Shanghai, China) for direct sequencing in both directions using the PCR primers.

### Phylogenetic analysis

Sequences were edited using DNAstar software (DNASTAR Inc., Madison, WI, USA), and BLAST (National Center of Biotechnology Information, NCBI) was used to determine related strains by identifying published sequences with high homology to the target sequence. Sequence alignment and phylogenetic analysis were performed using ClustalW and a neighbor-joining method using MEGA 5.0 (DNASTAR Inc.), respectively.

## Abbreviations

EDTA: Ethylene diamine tetraacetic acid
ITS-1: Specific internal transcribed spacer-1
PBS: Phosphate buffered saline
PCR: Polymerase chain reaction
RFLP: Restriction fragment-length polymorphism
VL: Visceral leishmaniasis

## References

[1] Alvar J, Yactayo S, Bern C (2006). Leishmaniasis and poverty. Trends Parasitol.

[2] Desjeux P (1996). Leishmaniasis. Public health aspects and control. Clin Dermatol.

[3] Boelaert M, Criel B, Leeuwenburg J, Van Damme W, Le Ray D, Van der Stuyft P (2000). Visceral leishmaniasis control: a public health perspective. Trans R Soc Trop Med Hyg.

[4] Guerin PJ, Olliaro P, Sundar S, Boelaert M, Croft SL, Desjeux P, Wasunna MK, Bryceson AD (2002). Visceral leishmaniasis: current status of control, diagnosis, and treatment, and a proposed research and development agenda. Lancet Infect Dis.

[5] de Vries HJ, Reedijk SH, Schallig HD (2015). Cutaneous leishmaniasis: recent developments in diagnosis and management. Am J Clin Dermatol.

[6] Lu HG, Zhong L, Guan LR, Qu JQ, Hu XS, Chai JJ, Xu ZB, Wang CT, Chang KP (1994). Separation of Chinese *Leishmania* isolates into five genotypes by kinetoplast and chromosomal DNA heterogeneity. Am J Trop Med Hyg..

[7] Wang JY, Cui G, Chen HT, Zhou XN, Gao CH, Yang YT (2012). Current epidemiological profile and features of visceral leishmaniasis in people's Republic of China. Parasit Vectors.

[8] Cao DP, Guo XG, Chen DL, Chen JP (2011). Species delimitation and phylogenetic relationships of Chinese *Leishmania* isolates reexamined using kinetoplast cytochrome oxidase II gene sequences. Parasitol Res.

[9] Yang BB, Guo XG, Hu XS, Zhang JG, Liao L, Chen DL, Chen JP (2010). Species discrimination and phylogenetic inference of 17 Chinese *Leishmania* isolates based on internal transcribed spacer 1 (ITS1) sequences. Parasitol Res.

[10] Guan LR (2009). Present situation of visceral leishmaniasis and prospect for its control in China. Chinese J Parasitol Parasit Dis.

[11] Guan LR, Shen WX (1991). Recent advances in visceral leishmaniasis in China. Southeast Asian J Trop Med Public Health.

[12] Zheng CJ, Xue CZ, Wu WP, Zhou XN (2017). Epidemiological characteristics of Kala-azar disease in China, during 2005–2015. Chinese J Epidemiol.

[13] Guan LR, Zuo XP, Yimamu (2003). Reemergence of visceral leishmaniasis in Kashi Prefecture, Xinjiang. Chinese J Parasitol Parasit Dis.

[14] Wang LY, Wu WP, Fu Q, Guan YY, Han S, Niu YL, Tong SX, Osman I, Zhang S, Kaisar K (2016). Spatial analysis of visceral leishmaniasis in the oases of the plains of Kashi Prefecture, Xinjiang Uygur Autonomous Region, China. Parasit Vectors.

[15] Bern C, Maguire JH, Alvar J (2008). Complexities of assessing the disease burden attributable to leishmaniasis. PLoS Negl Trop Dis.

[16] Mosleh IM, Geith E, Natsheh L, Abdul-Dayem M, Abotteen N (2008). Cutaneous leishmaniasis in the Jordanian side of the Jordan Valley: severe under-reporting and consequences on public health management. Tropical Med Int Health.

[17] Singh SP, Reddy DC, Rai M, Sundar S (2006). Serious underreporting of visceral leishmaniasis through passive case reporting in Bihar, India. Trop Med Int Health.

[18] Zijlstra EE (2016). Visceral leishmaniasis: a forgotten epidemic. Arch Dis Child.

[19] Wang JY, Gao CH, Yang YT, Chen HT, Zhu XH, Lv S, Chen SB, Tong SX, Steinmann P, Ziegelbauer K (2010). An outbreak of the desert sub-type of zoonotic visceral leishmaniasis in Jiashi, Xinjiang Uygur autonomous region, People's Republic of China. Parasitol Int.

[20] Fu Q, Wu WP, Tong SX, Israyil O, Zhang S, Iskender K (2009). Study on time-space clustering regarding the distribution of visceral leishmaniasis in Kashgar Region, Xinjiang. Chinese J Epidemiol.

[21] Bern C, Haque R, Chowdhury R, Ali M, Kurkjian KM, Vaz L, Amann J, Wahed MA, Wagatsuma Y, Breiman RF (2007). The epidemiology of visceral leishmaniasis and asymptomatic leishmanial infection in a highly endemic Bangladeshi village. Am J Trop Med Hyg.

[22] Topno RK, Das VN, Ranjan A, Pandey K, Singh D, Kumar N, Siddiqui NA, Singh VP, Kesari S, Kumar N (2010). Asymptomatic infection with visceral leishmaniasis in a disease-endemic area in Bihar, India. Am J Trop Med Hyg.

[23] Moreno I, Álvarez J, García N, de la Fuente S, Martínez I, Marino E, Toraño A, Goyache J, Vilas F, Domínguez L (2014). Detection of anti-*Leishmania infantum* antibodies in sylvatic lagomorphs from an epidemic area of Madrid using the indirect immunofluorescence antibody test. Vet Parasitol.

[24] Guan LR (1991). Current status of kala-azar and vector control in China. Bull World Health Organ.

[25] Del Rio L, Chitimia L, Cubas A, Victoriano I, De la Rúa P, Gerrikagoitia X, Barral M, Muñoz-García CI, Goyena E, García-Martínez D (2014). Evidence for widespread *Leishmania infantum* infection among wild carnivores in *L. infantum* periendemic northern Spain. Prev Vet Med.

[26] Kassiri H, Naddaf SR, Javadian EA, Mohebali M (2013). First report on isolation and characterization of *Leishmania major* from *Meriones hurrianae* (Rodentia: Gerbillidae) of a rural cutaneous leishmaniasis focus in South-Eastern Iran. Iranian Red Crescent Med J.

[27] Millán Javier, Ferroglio Ezio, Solano-Gallego Laia (2014). Role of wildlife in the epidemiology of Leishmania infantum infection in Europe. Parasitology Research.

[28] Donalisio MR, Paiz LM, da Silva VG, Richini-Pereira VB, von Zuben APB, Castagna CL, Motoie G, Hiramoto RM, Tolezano JE (2017). Visceral leishmaniasis in an environmentally protected area in southeastern Brazil: epidemiological and laboratory cross-sectional investigation of phlebotomine fauna, wild hosts and canine cases. PLoS Negl Trop Dis.

[29] Bhattarai NR, Van der Auwera G, Rijal S, Picado A, Speybroeck N, Khanal B, De Doncker S, Das ML, Ostyn B, Davies C (2010). Domestic animals and epidemiology of visceral leishmaniasis, Nepal. Emerg Infect Dis.

[30] Singh N, Mishra J, Singh R, Singh S (2013). Animal reservoirs of visceral leishmaniasis in India. J Parasitol.

[31] Gao CH, Wang JY, Zhang S, Yang YT, Wang Y (2015). Survey of wild and domestic mammals for infection with *Leishmania infantum* following an outbreak of desert zoonotic visceral leishmaniasis in Jiashi, People's Republic of China. PLoS One.

[32] Schonian G, Nasereddin A, Dinse N, Schweynoch C, Schallig HD, Presber W, Jaffe CL (2003). PCR diagnosis and characterization of *Leishmania* in local and imported clinical samples. Diagn Microbiol Infect Dis.

[33] Carvalho Ferreira AL, Carregal VM, de Almeida FS, Leite RS, de Andrade AS (2014). Detection of *Leishmania infantum* in 4 different dog samples by real-time PCR and ITS-1 nested PCR. Diagn Microbiol Infect Dis.

[34] Stauch A, Sarkar RR, Picado A, Ostyn B, Sundar S, Rijal S, Boelaert M, Dujardin JC, Duerr HP (2011). Visceral leishmaniasis in the Indian subcontinent: modelling epidemiology and control. PLoS Negl Trop Dis.

[35] Tiwary P, Singh SS, Kushwaha AK, Rowton E, Sacks D, Singh OM, Sundar S, Lawyer P (2017). Establishing, expanding, and certifying a closed colony of *Phlebotomus argentipes* (Diptera: Psychodidae) for xenodiagnostic studies at the kala azar medical research center, Muzaffarpur, Bihar, India. J Med Entomol.

[36] Esteva L, Vargas C, Vargas de León C (2017). The role of asymptomatics and dogs on leishmaniasis propagation. Math Biosci.

[37] Liao LF, Yan SS, Wu SBT, Wu M, Xu B, Zhang Y, Hou YY, Lei G. The first isolation of *Leishmania infantum* from *Lepus yarkandensis*. Chinese J Vect Biol Contr. 2009:45–7 [Article in Chinese].

[38] Guan LR, Cai CJ, Zuo XP (1999). Progress of biological study on sandflies in Xinjiang Uygur autonomous region, China. End Dis Bull.

[39] Gu DA, Zuo XP, Osman Y, Lan QX, Jin CF, Zhou XJ, Wei F, Zhang Y (2010). Investigation on transmitting vectors of visceral leishmaniasis in Jiashi County, Xinjiang. Chinese J Parasitol Parasit Dis.

[40] Srividya G, Kulshrestha A, Singh R, Salotra P (2012). Diagnosis of visceral leishmaniasis: developments over the last decade. Parasitol Res.

[41] Georgiadou SP, Makaritsis KP, Dalekos GN (2015). Leishmaniasis revisited: current aspects on epidemiology, diagnosis and treatment. J Transl Int Med.

[42] Maia Z, Lírio M, Mistro S, Mendes CM, Mehta SR, Badaro R (2012). Comparative study of rK39 *Leishmania* antigen for serodiagnosis of visceral leishmaniasis: systematic review with meta-analysis. PLoS Negl Trop Dis.

[43] Guan LR, Qu JQ, Cai JJ, Matsumoto Y, Chang KP (2001). Detection of canine visceral leishmaniasis by rK 39 antigen dipstick method. Chinese J Parasitol Parasit Dis.

[44] Matlashewski G, Das VN, Pandey K, Singh D, Das S, Ghosh AK, Pandey RN, Das P (2013). Diagnosis of visceral leishmaniasis in Bihar India: comparison of the rK39 rapid diagnostic test on whole blood versus serum. PLoS Negl Trop Dis.

[45] Molinet FJ, Ampuero JS, Costa RD, Noronha EF, Romero GA (2013). Specificity of the rapid rK39 antigen-based immunochromatographic test Kalazar detect(r) in patients with cutaneous leishmaniasis in Brazil. Mem Inst Oswaldo Cruz.

[46] Qu JQ, Guan LR, Shulidan I, Zuo XP, Chai JJ, Chen SB, Kawazu S, Katakura K, Matsumoto Y, Reed SG (2000). Rapid screening with a recombinant antigen (rK39) for diagnosis of visceral leishmaniasis using dipstick. Chinese J Parasitol Parasit Dis.

[47] Regina-Silva S, Fortes-Dias CL, Michalsky ÉM, França-Silva JC, Quaresma PF, da Rocha Lima AC, Teixeira-Neto RG, Dias ES (2014). Evaluation of parasitological examination, kDNA polymerase chain reaction and rK39-based immunochromatography for the diagnosis of visceral leishmaniasis in seropositive dogs from the screening-culling program in Brazil. Rev Soc Bras Med Trop.

[48] Leite RS, de Almeida FS, Ituassu LT, de Melo MN, de Andrade AS (2010). PCR diagnosis of visceral leishmaniasis in asymptomatic dogs using conjunctival swab samples. Vet Parasitol.

[49] Pilatti MM, de Almeida FS, de Melo MN, de Andrade AS (2009). Comparison of PCR methods for diagnosis of canine visceral leishmaniasis in conjunctival swab samples. Res Vet Sci.

[50] Ajaoud M, Es-sette N, Hamdi S, El-Idrissi AL, Riyad M, Lemrani M (2013). Detection and molecular typing of *Leishmania tropica* from *Phlebotomus sergenti* and lesions of cutaneous leishmaniasis in an emerging focus of Morocco. Parasit Vectors.

[51] Muccio D, Veronesi F, Antognoni MT, Onofri A, Piergili Fioretti D, Gramiccia M (2012). Diagnostic value of conjunctival swab sampling associated with nested PCR for different categories of dogs naturally exposed to *Leishmania infantum* infection. J Clin Microbiol.

